# Supplementary figures and images for: Salidroside, 8(E)-Nuezhenide, and Ligustroside from Ligustrum japonicum Fructus Inhibit Expressions of MMP-2 and -9 in HT 1080 Fibrosarcoma
Source: Int J Mol Sci. 2022 Feb 28;23(5):2660. doi: 10.3390/ijms23052660 (PMC8910403; doi:10.3390/ijms23052660)

$^1\text{H}$  NMR and  $^{13}\text{C}$  NMR spectra of salidroside (SAL)

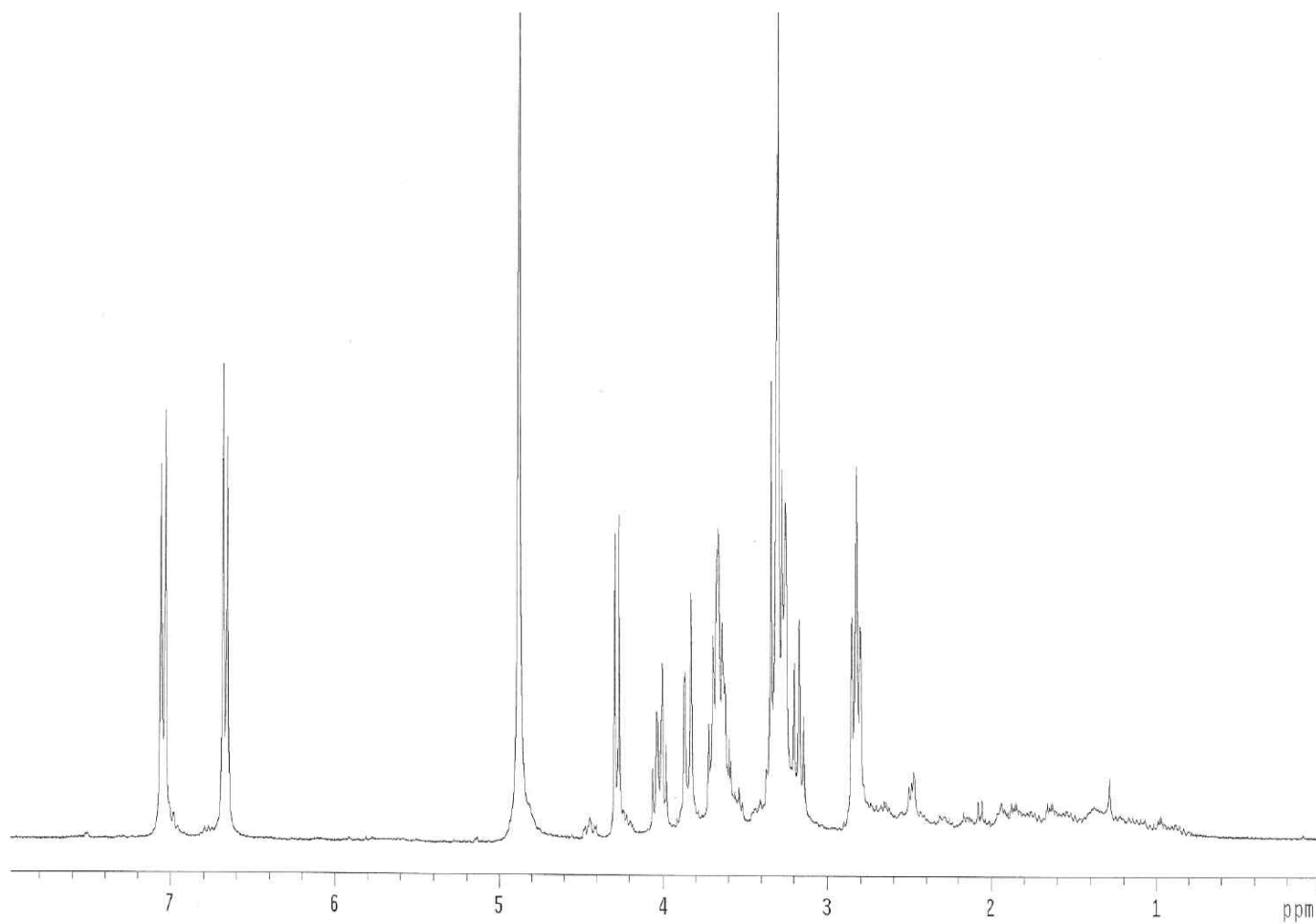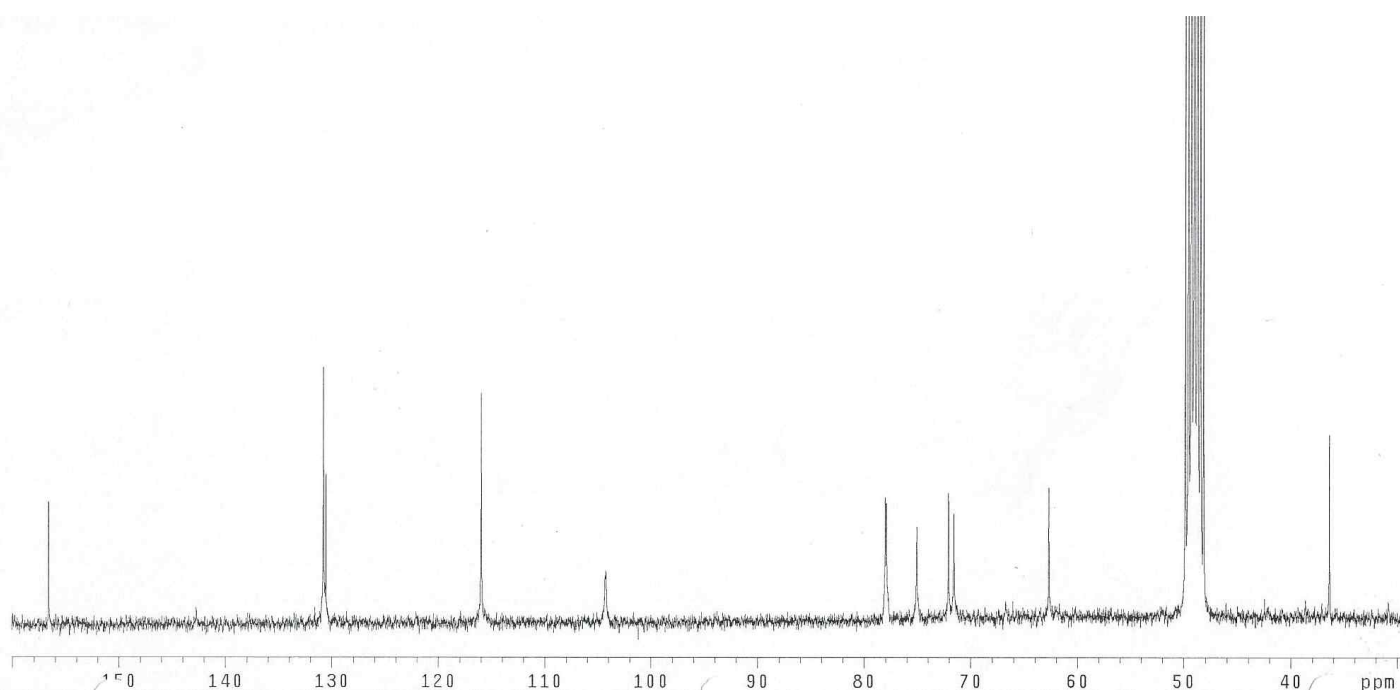

Supplement: Supplementary file 1 [file ijms-23-02660-s001.zip › Figure S1.pdf]

$^1\text{H}$  NMR and  $^{13}\text{C}$  NMR spectra of 8(E)-nuezhenide (**NZD**)

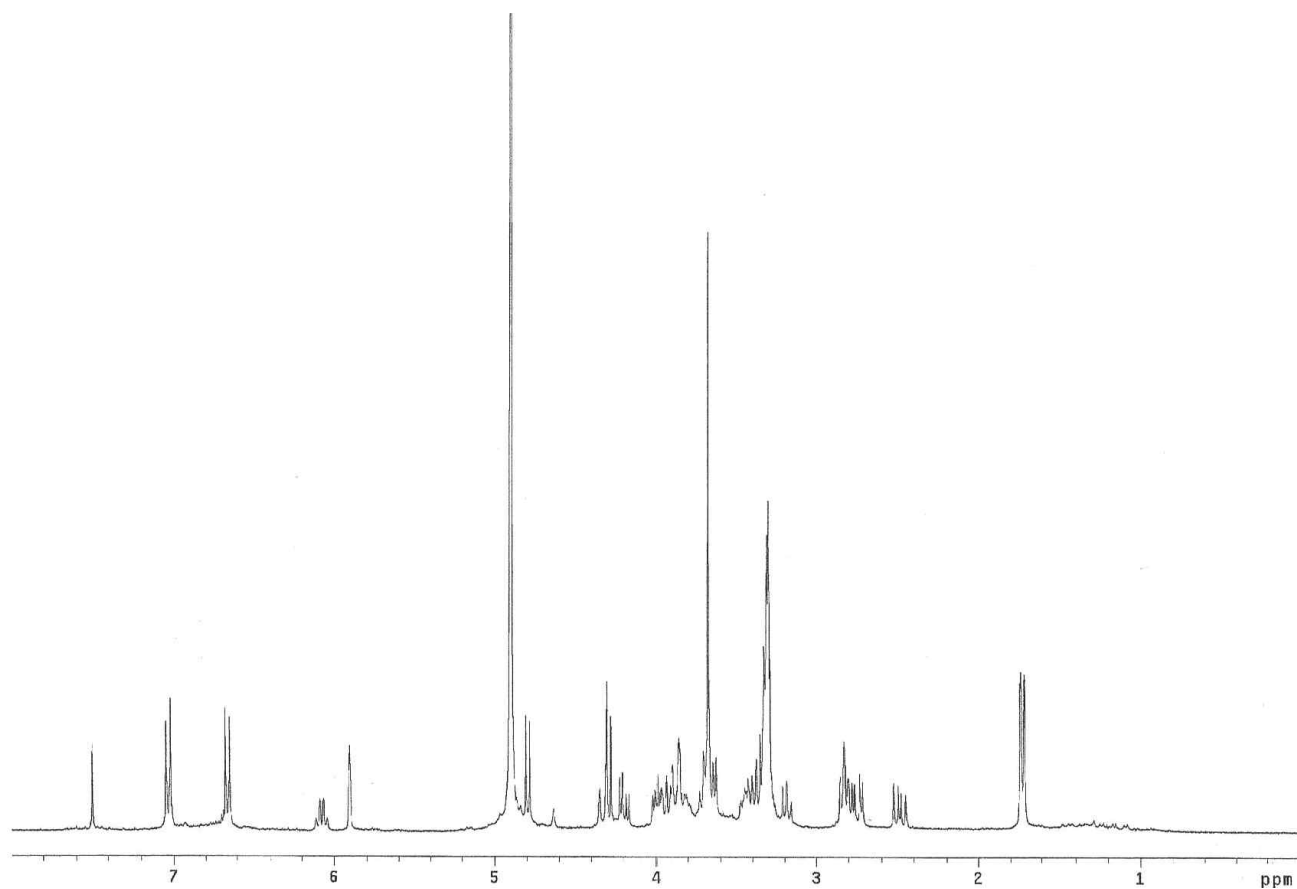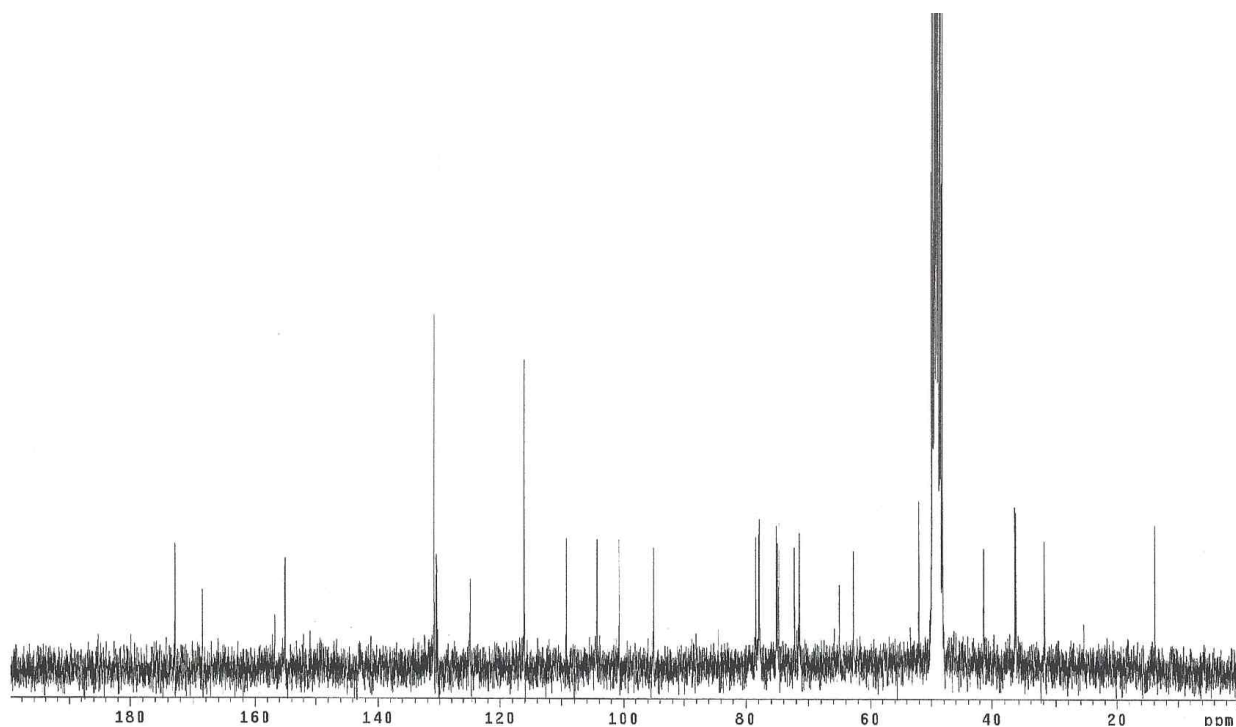

Supplement: Supplementary file 1 [file ijms-23-02660-s001.zip › Figure S2.pdf]

$^1\text{H}$  NMR and  $^{13}\text{C}$  NMR spectra of 8(E)-ligustroside (LIG)

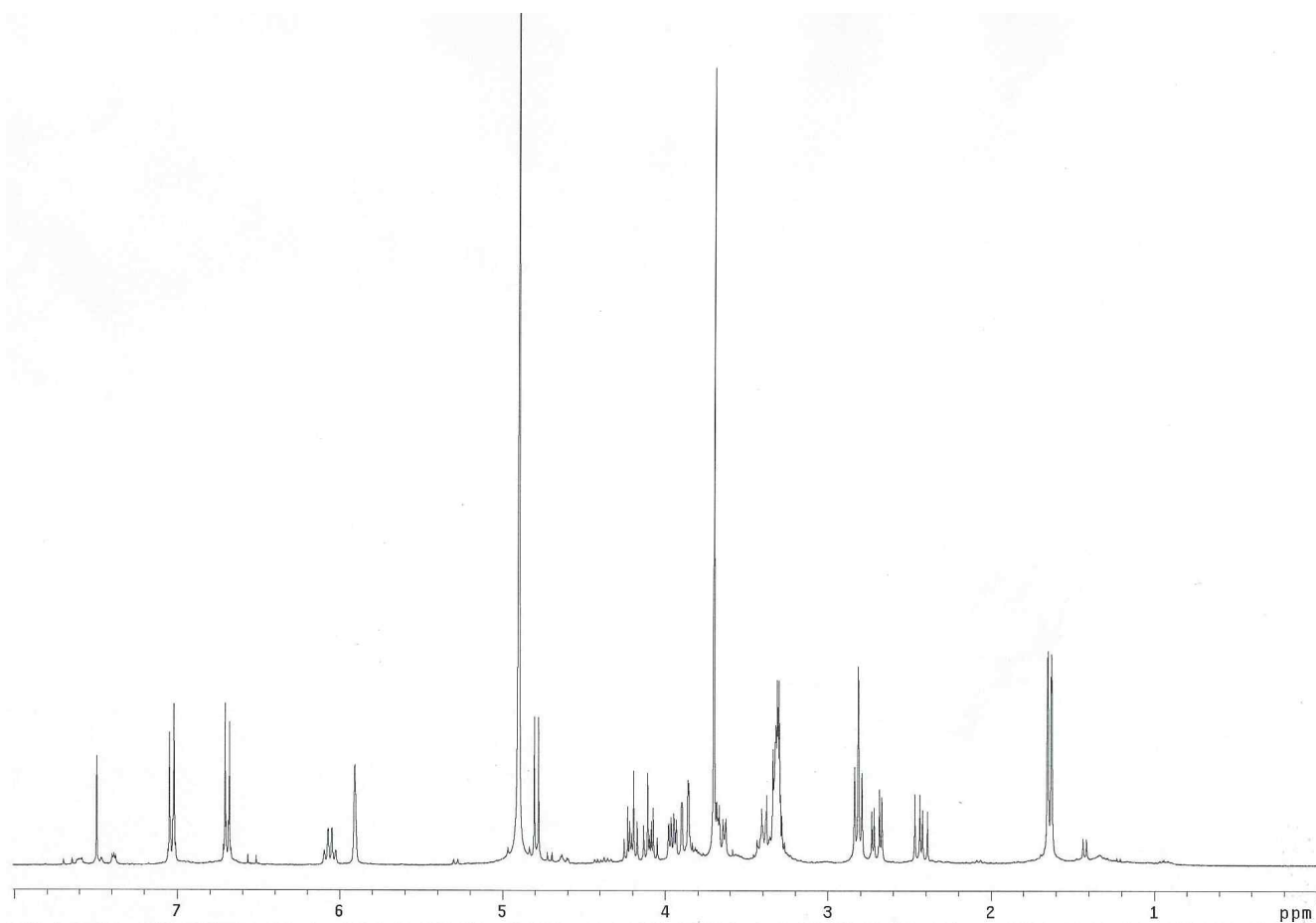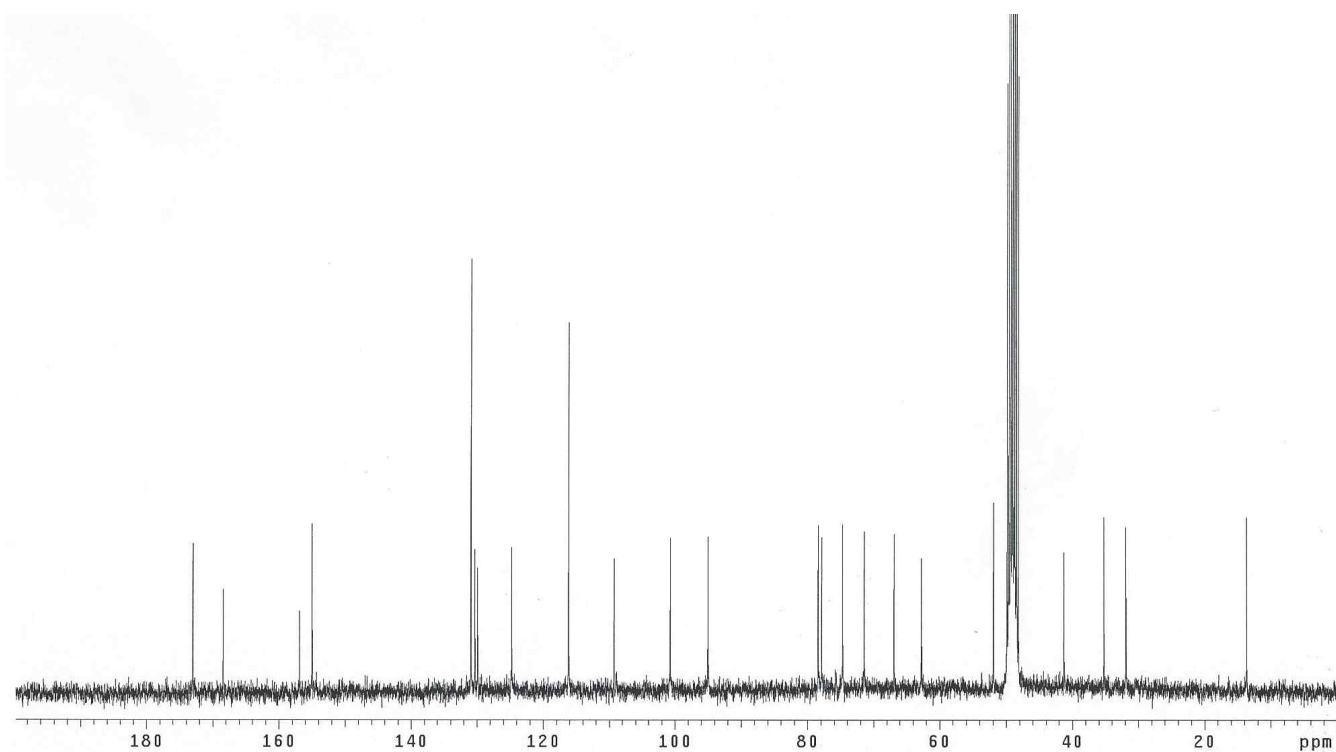

Supplement: Supplementary file 1 [file ijms-23-02660-s001.zip › Figure S3.pdf]
